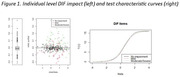# Utility of the PROMIS Physical Function measure using differential item functioning among individuals with cognitive impairment

**DOI:** 10.1002/alz.089039

**Published:** 2025-01-09

**Authors:** Rebecca Lovett, Andrea Russell, Abigail Vogeley, Morgan Bonham, Eileen Graham, Daniel Mroczek, Julia Yoshino‐Benavente, Rachel O'Conor, Lauren Opsasnick, Stephanie Batio, Michael S Wolf

**Affiliations:** ^1^ Northwestern University, Chicago, IL USA; ^2^ Northwestern University Feinberg School of Medicine, Chicago, IL USA

## Abstract

**Background:**

Impaired functional status is a central diagnostic feature of Alzheimer’s disease and related dementias (ADRD). Informant reporting is often relied upon, given concerns surrounding the ability of persons with ADRD to validly self‐report symptoms. We sought to investigate how cognitive impairment severity impacts psychometric properties of the Patient‐Reported Outcomes Measurement Information System Physical Function (PROMIS‐PF) scale.

**Methods:**

Data from 396 older adults participating in a longitudinal cohort study on aging was used for this analysis. Cognitive function was assessed using an extensive neuropsychological battery consisting of 13 tests across 5 cognitive domains; z‐scores were calculated for each test and used to determine cognitive impairment severity. Physical function was measured using the PROMIS‐PF short‐form 10a. Differential item functioning (DIF) analyses were conducted using the *lordif* package in R with Monte Carlo simulations.

**Results:**

Participants were on average 71.2 years old (SD 5.3), primarily female (72.0%), white (54.3%), and well‐educated (60.0% at least some college). Nearly two‐thirds were cognitively normal (72.5%), while 14.4% and 13.4% had a mild and moderate/severe cognitive impairment, respectively. Seven out of 10 PROMIS‐SF items were flagged for DIF. Direction of DIF varied; participants with mild impairment exhibited a tendency to underestimate their physical function, while those with moderate/severe impairment were characterized by a tendency to overestimate. McFaddens pseudo R^2^ effect sizes were all below published thresholds of 0.13 (range: 0.01‐0.05). Test characteristic curves (TCC) were overlapping between the 3 groups.

**Conclusions:**

Findings indicate the presence of DIF by cognitive impairment severity on the PROMIS‐PF measure, although the magnitude of effect and overlapping TCC curves suggest the observed DIF was negligible. Nevertheless, researchers and clinicians should be aware that individuals with a cognitive impairment may respond differently to certain items on this measure and further study is needed to evaluate measurement bias among patients with cognitive impairment, including ADRD. However, overall, our evidence suggests patients with varying levels of cognitive impairment can provide reliable estimates of physical function using this measure. This has implications for researchers and clinicians seeking to assess functional status among older adults with ADRD, particularly if supplemental reports of functional status are not feasible or unavailable.